# Carbon nanotubes for wound healing: material design, mechanistic insights

**DOI:** 10.3389/fbioe.2025.1732037

**Published:** 2026-01-12

**Authors:** Nidhi Poddar, Kaustubh Naik, Sushma Kumari

**Affiliations:** 1 Centre for Biomaterials, Cellular and Molecular Theranostics, Vellore Institute of Technology, Vellore, Tamil Nadu, India; 2 Department of Cell Biology and Physiology, Washington University School of Medicine, St. Louis, MO, United States

**Keywords:** carbon nanotubes, conductive, hydrogel, wound healing, scaffolds, angiogenesis

## Abstract

Chronic wounds such as diabetic ulcers remain significantly higher in global healthcare burden due to impaired angiogenesis, infection, and sustained inflammation. Carbon nanotubes (CNTs) are promising candidates for advanced wound-dressing applications due to their exceptional electrical conductivity, high mechanical strength, photothermal performance, and ease of surface modification. This review discusses recent progress in their functions in haemostasis, microbial protection, anti-inflammatory regulation, and tissue repair. We discussed research papers on CNT-based multifunctional hydrogels, electrospun scaffolds, and innovative dressings for bioactive agent delivery, electrical stimulation, and real-time monitoring of wound healing. We also discussed *in vivo* preclinical studies demonstrating significant re-epithelialization and increased angiogenesis, with accelerated wound closure in disease-impaired healing models, such as diabetes. Nevertheless, limitations such as cytotoxicity, impediments to scale-up manufacturing, and regulatory issues hinder direct clinical translation. To overcome these drawbacks, several approaches, such as chemical functionalization, biodegradable CNT derivatives, and hybrid nanocomposites, have been developed. Finally, we describe the translational path for CNT-based wound-healing applications and offer perspectives on future therapeutic interventions for chronic and complex wounds in the context of precision medicine.

## 1 Introduction

**FIGURE 1:**
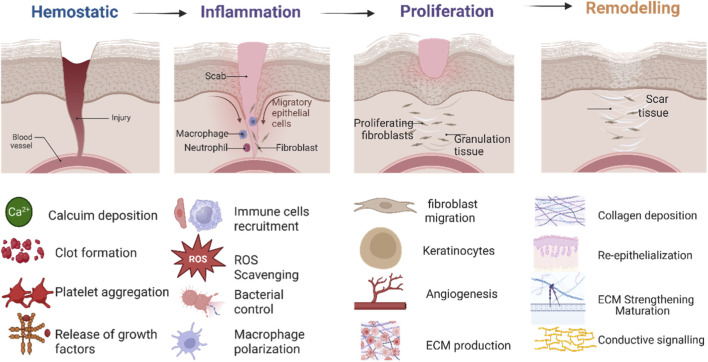
Schematic representation of the sequential phases of wound healing and their cellular-molecular regulation. The hemostatic phase involves platelet aggregation, calcium deposition, clot formation, and growth factor release. The inflammatory phase is characterized by neutrophil and macrophage recruitment, ROS generation, bacterial clearance, and macrophage polarization. The next phase involves proliferating cells, such as fibroblasts, keratinocytes, and endothelial cells, which initiate the formation of granulation tissue and angiogenesis. Finally, the remodeling phase includes collagen deposition, ECM maturation, and restoring tensile integrity.

Wound healing is a well-coordinated, dynamic biological process in which tissue integrity and function are restored after injury. It sequentially follows four overlapping phases, in which the time-line between haemostasis and remodelling is distinct (Wilkinson and Hardman, 2020), as shown in Figure 1. The haemostatic phase begins immediately after trauma, prevents blood loss via clot formation, and forms a provisional matrix for cell infiltration, thereby promoting healing. This is followed by the inflammatory phase, during which neutrophils and macrophages clear pathogens and debris while releasing cytokines and growth factors to facilitate repair. The proliferative phase, marked by the recruitment and activation of fibroblasts, endothelial cells, and keratinocytes, controls events such as granulation tissue formation, angiogenesis, fibroplasia, and re-epithelialization, leading to wound healing. In the final remodeling or maturation phase, the extracellular matrix (ECM) is strengthened and restructured. During the maturation phase, type III collagen is gradually replaced by type I collagen, restoring tensile strength and functional capacity to the repaired tissue. Although traditionally described as separate stages, wound healing is, in reality, a continuous, overlapping sequence of events that varies with tissue type and differs significantly between acute and chronic wounds (Gonzalez et al., 2016). Acute wounds usually follow their normal physiological course of healing, with distinct stages, and finally restore tissue integrity within a specified time. In these situations, successful cross-talk among immune cells, fibroblasts, and endothelial cells is essential for timely clotting of the wound, as well as for collagen deposition and re-epithelialization. Chronic wounds, including diabetic ulcers, pressure sores, and leg ulcers, are frequently associated with chronic inflammation, impaired angiogenesis, and microbial infection, which is challenging to treat with conventional methods.

The conventional method of wound care involves topical antimicrobial ointments, dressings, or systemic pharmacological agents, with primarily symptom-based rather than biological barrier-destructive effects (Pereira and Bártolo, 2016). Recently, nanotechnology has emerged as a transformative tool for redefining treatment options in biomedicine by developing new products/methods for diagnostics, drug delivery, tissue engineering, and regenerative medicine. This technique is believed to be effective because of its interdisciplinary approach at the nanoscale (1 nm–100 nm) and its ability to enhance size-dependent physicochemical characteristics that are often different from those of bulk materials. In this context, various nanosystems, such as nanoparticles, nanoemulsions, nanocomposites, and nanotubes, have been examined to enhance drug targeting at the wound site, minimize systemic side effects, and improve therapeutic activity (Sangnim et al., 2024). Among various nanomaterials, carbon-based nanomaterials (including CNTs) are particularly interesting due to their excellent versatility and efficiency in tissue regeneration (Sridhar et al., 2024).

CNTs are the graphene sheets that are rolled and form nanotubes themselves, appearing as single-walled carbon nanotubes (SWCNTs) diameter 0.4–2 nm) or multi-walled nanotubes (MWCNTs) with a diameter up to ∼100 nm (Thakur et al., 2022) (Sayed and Shaikh, 2024). The significance of CNTs in wound repair has primarily been attributed to their outstanding mechanical properties, including tensile strengths of 10–100 GPa and Young’s moduli of 1 TPa. CNTs exhibit superior performance compared with most traditional materials and can form strong, tissue-like scaffolds that promote cell adhesion and migration (Holmannova et al., 2022). Electrically conductive CNTs up to 10^6^ S/m promote bioelectrical signalling, influencing cellular behavior in tissues such as skin. Additionally, high thermal conductivity and NIR absorptivity enable the photothermal effect, in which localized heating destroys bacterial membranes with less cytotoxicity to host cells. Furthermore, the availability of a large surface area (up to 1,600 m^2^/g) allows for high-capacity loading of drugs such as antibiotics, growth factors, or antioxidants with a controlled drug-releasing profile regulated by pH, temperature, or light. Furthermore, functionalization approaches, including the covalent addition of carboxyl, hydroxyl, or amine groups, or non-covalent wrapping with polymers such as PEG, can make the materials more biocompatible and/or soluble, promote selective delivery, and alleviate some of the potential toxicity of bare CNTs (Madaninasab et al., 2025), Figure 2.

**FIGURE 2:**
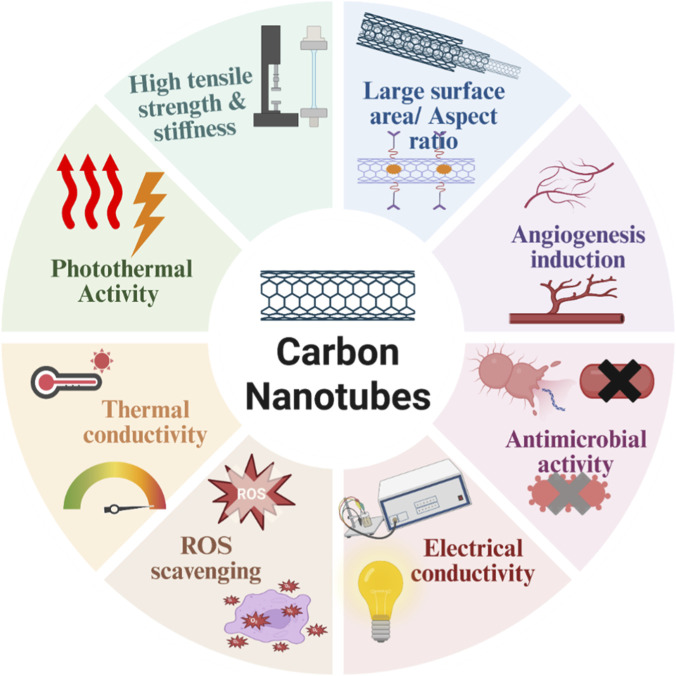
Distinctive physicochemical and biological characteristics of CNTs for wound healing. CNTs have high tensile strength and rigidity, making them suitable for reinforcing scaffolds with greater surface area/aspect ratio, which would afford high drug/gene loading. The electrical conductivity of CNTs enables electrostimulation-induced tissue repair, while their thermal and photothermal conductivities help with infection management and on-demand release. Biological activities include antimicrobial activity, ROS sequestration, and angiogenesis induction, establishing CNTs as a multifunctional nanomaterial for advanced wound treatment.

The significant antimicrobial and immunomodulatory properties of CNTs have tackled key challenges in wound management. The sharp, needle-like structures of CNTs allow mechanical disruption of bacterial membranes, and their surface chemistry promotes the generation of reactive oxygen species (ROS), resulting in intense, broad-spectrum antibacterial activity, even against resistant strains, during the wound-healing process or those formed by biofilm-forming pathogens.

CNTs may also control wound inflammation by scavenging excessive ROS, suppressing pro-inflammatory cytokines, and guiding macrophage polarization toward an anti-inflammatory phenotype, thus providing a microenvironment that favours accelerated tissue repair (Zarouki et al., 2025). During the proliferative phase, CNT composites promote fibroblast proliferation, collagen synthesis, and angiogenesis through activation of pathways such as PI3K/Akt or ERK/MAPK, sometimes assisted by electrical stimulation or the inclusion of bioactive molecules (Liu and Fang, 2025). For instance, in a preclinical experiment with CNT-drug-loaded hydrogels, Zhang et al. and their research team achieved 99% healing of diabetic chronic wounds in rodent models after 14 days (Zhang et al., 2023).

From 2020 to 2025, remarkable developments were made in wound care applications, including CNT-based electrically conductive photothermal and antibacterial scaffolds and functionalized CNTs for targeted drug release. Integration of CNTs with chitosan, PLA, and other biopolymers resulted in nanofibrous dressings with enhanced mechanical strength and antioxidant activity, thereby facilitating faster wound healing in burn and surgical wounds (Vardharajula et al., 2012) (Sayed and Shaikh, 2024). Patenting activity indicates the worldwide attention towards developing biodegradable CNT matrices and advanced dressings with sensors. Nevertheless, several barriers remain, including impurity-mediated toxicity, aggregation-induced inflammation, and the lack of scalable, standardized production.

In this review, we start with the structure, classification, and essential properties of CNTs, and summarize their *in vitro* and *in vivo* behaviour related to biological activities associated with wound healing. We review *in vitro* and *in vivo* models that have been utilized as preclinical tools for testing of CNT-based therapeutics, and the broad use of these exciting nanotubes in diverse biomedical applications such as haemostasis, antibacterial or anti-inflammatory systems, diabetic wound healing and angiogenesis, tissue engineering scaffolds, smart-CNT enabled dressings, sensors, and burn-care technologies. The review also explores clinical translation, addressing current advancements, regulatory barriers, and potential solutions to overcome these challenges, and concludes with an outlook for the development of CNT-based wound management technologies.

## 2 Overview, structure, types, and properties of carbon nanotubes

CNT research began with the pioneering discovery of multiwalled CNTs (MWCNTs) by Sumio Iijima in 1991, followed soon after by the identification of single-walled CNTs (SWCNTs). Since then, CNTs have attracted significant attention due to their inherent properties, including mechanical reinforcement, structural diversity, hydrophobicity, flexibility, and electrical conductivity, which make them suitable for applications ranging from nanoelectronics to drug delivery (Roldo and Fatouros, 2013) (de Andrade et al., 2024) (Rathinavel et al., 2021).

**FIGURE 3:**
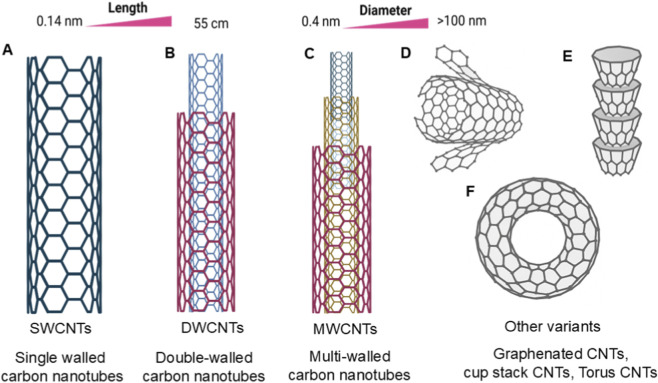
Structural representation of **(A)** single walled (SW), **(B)** double walled (DW), **(C)** multi-walled (MW) carbon nanotubes, and **(D)** graphenated CNTs, **(E)** cup stack CNTs resemble stacked cones, and **(F)** torus CNTs are donut-shaped.

CNTs belong to the fullerene structural family and are a third form of carbon (diamond and graphite being the other two). CNTs are seamless cylinders comprising sp^2^-hybridized carbon and can be rolled up to form a hexagonal lattice with superb mechanical, electrical, and thermal properties (Wang et al., 2024a) (Jia et al., 2024). The structural orientation of carbon nanotubes (CNTs) can adopt different configurations, such as armchair, zigzag, or chiral, which strongly influences their electronic nature, ranging from metallic to semiconducting (Ijaz et al., 2023). Based on the number of layers, CNTs are typically divided into SWCNTs and MWCNTs, as shown in Figure 3.

### 2.1 Single-walled carbon nanotubes (SWCNTs)

SWCNTs are formed from a single sheet of graphene rolled into a tube, with diameters usually between 0.4 and 2 nm. Their electrical properties are strongly influenced by their chirality whether the tube is in an armchair, zigzag, or chiral configuration, which determines whether they behave as metals or semiconductors. One of the most notable features of SWCNTs is their exceptionally high surface area, which can reach ∼1,300 m^2^/g. This large surface provides abundant sites for drug loading and bioconjugation. The complexes formed when used as drug carriers exhibit prolonged retention in systemic circulation compared with free drugs, enabling sustained cellular uptake via the enhanced permeability and retention (EPR) effect (Hughes et al., 2024).

Upon targeted delivery, functionalized SWCNTs gradually release their drug payload at the desired site. They are subsequently cleared from the body through the biliary system and excreted in feces. These findings highlight the potential of SWCNTs as efficient nanocarriers, making them promising platforms for drug delivery and wound-healing applications (Anzar et al., 2020).

### 2.2 Multiwalled carbon nanotubes (MWCNTs)

MWCNTs are formed as coaxial cylinders, consisting of several concentric layers of graphene sheets wrapped into the shape of a tube-within-a-tube. They generally have outer diameters of 2–100 nm and inner diameters of 1–3 nm. The length of such nanotubes ranges from several nanometres to a few micrometres, depending on the fabrication technique. The multilayered structure of MWCNTs also makes them mechanically stronger and stiffer than SWCNTs (Rahamathulla et al., 2021). Therefore, they are suitable candidates for applications that require mechanical reinforcement, such as scaffolds in tissue engineering and wound healing. The hollow core with many sidewalls provides high load capacity for therapeutic molecules, making them well-suited as potential drug-delivery carriers. When loaded into biopolymeric scaffolds composed of chitosan, gelatin, alginate, and other biomaterials, MWCNTs instantly enhance cellular adhesion, viability, and proliferation while reducing cytotoxicity (Alhashmi Alamer and Almalki, 2022).

### 2.3 Other variants

In addition to the classical SWCNTs and MWCNTs, several less conventional structural variants of CNTs have been reported, each offering unique physicochemical features. Graphenated CNTs (g-CNTs) consist of CNT backbones decorated with graphene layers, thereby significantly increasing their surface area and electron-transfer efficiency, making them attractive for electrochemical and biomedical applications (Eatemadi et al., 2014). Cup-stacked CNTs are another variant, composed of truncated-cone-like segments stacked. These structures have thousands of open edges and defect sites, providing a large number of chemical functionalization sites and, in turn, excellent reactivity (Kim et al., 2024). CNT torus (looped, ring) has been reported. This exotic shape endows unique magnetic and electronic properties, which can have broader applications in CNT-based high-end nanodevices and bioelectronics. In aggregate, these structural changes extend the utility of CNTs, enabling the engineering of desired properties for specific biomedical and technological applications (Thakur et al., 2022).

### 2.4 Properties of CNTs

CNTs possess a high surface area and modifiable surface functionalization, which are beneficial for drug delivery, biosensing, antimicrobial agents, and wound healing. MWCNTs are rigid, multifunctional materials that are particularly appealing for biomedical applications (e.g., fields that require high strength and bioactivity) (Thakur et al., 2022). The chirality and conformations of CNTs strongly affect their electrical properties: armchair CNTs are metallic, while zigzag and chiral CNTs may be semiconductors. This electrical tunability renders CNTs highly attractive for future materials in electronics and biomedical applications (Costa et al., 2016).

In addition, CNTs are also well known for their outstanding mechanical strength (tensile strength up to 10–100 GPa and a Young’s modulus of nearly 1 TPa), enabling them to withstand high levels of strain without deformation failure (Nurazzi et al., 2021). They also have high thermal conductivity (2000–3000 W/mK), outstanding electrical conductivity (10^6^–10^7^ S/m). Another key feature is their extremely high surface area-to-volume ratio, which would further facilitate interaction with biomolecules and cells. Due to their high aspect ratio (>100), they can be used to create conductive percolation networks in polymer composites at very low loading levels (0.1%–1%), thereby substantially increasing the material’s electrical conductivity (Li et al., 2007). These characteristics make them suitable for use in efficient heat dissipation and electrical charge transfer applications. These distinct physicochemical attributes of CNTs set them apart from other nanomaterials and are responsible for their growing importance in biomedicine, energy technologies, sensing platforms, and catalytic systems (Gupta et al., 2019).

Despite CNTs’ inherent hydrophobicity and tendency to aggregate, biomolecule functionalization can significantly enhance dispersion, biocompatibility, and drug-loading capacity. In biomedical engineering, the exceptional properties of CNTs are considered particularly useful and have been used to fabricate composites with natural polymers such as alginate, collagen, gelatine, and chitosan, or synthetic polymers, to reinforce the mechanical strength of scaffolds, enhance electrical stimulation, and improve cell interactions (Murjani et al., 2022). Chemical or surface modifications have enabled CNTs to serve as multifunctional platforms in the biomedical field, providing a large surface area and tunable surface chemistry for drug loading (accessible adsorption sites) and structural support from their mechanical strength (Islam et al., 2022). Their excellent electrical properties have also been used to induce cellular responses, especially in electrically active tissues, including neural, cardiac, and muscular tissues, in which endogenous signals for cell-cell communication are essentially electrical (Nekounam et al., 2021).

These features render CNTs highly promising in biomedicine, including drug delivery, biosensing, tissue engineering, and wound healing. CNTs possess distinctive physicochemical properties and a broad range of biomedical applications. Their unique combination of mechanical strength, electrical conductivity, and controllable surface chemistry makes them among the most widely investigated nanomaterials in regenerative medicine, drug delivery, and wound healing. With advances in functionalization and toxicity reduction, CNTs are gradually moving towards translational biomedical applications (Gupta et al., 2019).

## 3 Biological activities relevant to wound healing

Wound healing is a complex series of overlapping phases-hemostasis, inflammation, proliferation, and remodelling that involves precisely regulated cellular, molecular, and biochemical events (Sen, 2019). Disruption of any phase, such as persistent inflammation in diabetic wounds or microbial colonization in chronic ulcers, can compromise healing and lead to non-healing states (Darwin and Tomic-Canic, 2018). Thus, materials for wound healing need to interact with this complex environment in numerous ways, including promoting each phase, such as hemostasis, managing infection, influencing inflammation, and inducing post-traumatic tissue regeneration. CNTs have various biological outcomes closely associated with these processes owing to their nanoscale size, surface chemistry, and multifunctional nature (Sharma et al., 2016). Unlike conventional dressings, which are passive barriers to synergies, CNT-based systems become active players within the wound microenvironment and interact with platelets, immune cells, fibroblasts, keratinocytes, endothelial cells, and microbes present at the wound site. Understanding these biological interactions is essential for understanding the therapeutic potential of CNTs in wound healing (Nie et al., 2023).

CNTs can modulate biological events in wound healing. A very early phase of wound healing is hemostasis, in which blood clotting and the formation of a fibrin plug prevent excessive bleeding and provide a provisional matrix for cellular infiltration. The high surface roughness and fibrous architecture of CNTs replicate the features of extracellular matrix (ECM) proteins that promote platelet adhesion and activation. Numerous experimental studies have shown that CNT dressings promote clotting more efficiently than standard gauze (Madaninasab et al., 2025). The nanotopography might increase platelet aggregation and stimulate thrombin generation, resulting in accelerated fibrin deposition. Furthermore, CNTs incorporated into polymer hydrogels or sponges showed decreased bleeding time in animal models, suggesting a potential use as hemostatic materials (Zhao et al., 2018) (Patil et al., 2021). Rapid hemostasis is critical in trauma or surgical settings, as timely control of bleeding can be lifesaving. The hemostatic enhancement of CNTs is therefore a crucial platform for their application in multifunctional wound dressings.

In addition to blood clotting, another primary concern in wound healing is preventing microbial infection. Bacterial colonization not only retards subsequent healing but also causes chronic inflammation and necrosis. CNTs have intrinsic antimicrobial activity that works through various mechanisms (Baek et al., 2019). The needle-like sharpness permits direct physical penetration and disruption of the bacterial membrane, leading to leakage of cytoplasmic contents and subsequent cell death. Also, CNTs are capable of generating (spontaneously and/or in light contact) reactive oxygen species (ROS), which may lead to bacterial oxidative stress. Moreover, CNTs exhibit a selective near-infrared (NIR) absorption feature that can be converted into local heat, enabling photothermal antibacterial applications (Hadidi and Mohebbi, 2022). CNTs under NIR irradiation incorporated into a hydrogel or film can achieve local heating to bactericidal levels, eliminating bacteria and preventing biofilm formation (Zhong et al., 2022). Significantly, this photothermal modality provides a non-antibiotic treatment option against infections, minimizing the potential risk of antibiotic resistance. Upon combination with antimicrobial peptides (AMPs) and conventional antibiotics, including silver nanoparticles, a synergistic effect was observed, yielding robust, multi-layer coverage against infection when CNTs were utilized (Wang et al., 2024b).

Although it is essential to control infection, excessive and unresolved inflammation poses another significant barrier to wound healing, particularly in chronic diabetic ulcers. CNTs have been shown to regulate the immune response during healing favourably. Studies suggest that functionalized CNTs influence macrophage polarization, shifting them from the pro-inflammatory M1 phenotype toward the pro-healing M2 phenotype. This shift is associated with decreased release of pro-inflammatory cytokines such as TNF-α and IL-6, on the one hand, and increased expression of anti-inflammatory factors such as IL-10 and TGF-β (Louiselle et al., 2021). Such immunomodulation helps resolve the inflammatory phase and subsequently allows progression to the proliferative healing phase. In addition, CNTs appear to mitigate oxidative stress in host tissues by scavenging excessive ROS, which is especially useful for wounds with a high burden of oxidative stress that leads to continuous damage during the repair process. These two divergent functions, killing them with ROS in microbes and moderating ROS in host cells, make up just two examples of the many biological interactions that CNTs undertake (Lim et al., 2023) (Meng et al., 2015).

As the wound progresses into the proliferative phase, cellular proliferation, angiogenesis, and extracellular matrix deposition predominate. CNTs also interact positively with fibroblasts and keratinocytes, which are crucial cells during this phase. The nanofibrous architecture provides a scaffold-like structure that enhances fibroblast adhesion, spreading, and growth. When embedded within composite hydrogels or nanofibrous mats, CNTs stimulate fibroblast-derived collagen synthesis and thus expedite granulation tissue formation. Keratinocytes, which play a key role in re-epithelialisation, also showed increased migration and proliferation on CNT-modified substrates. The conductive properties of CNTs may also play a role, as bioelectrical cues influence keratinocyte migration during epithelial sheet closure. Electrical stimulation supplied by CNT-based dressings has been reported to augment such processes, illustrating the intertwining of material attributes with biological signalling (Zhang et al., 2021).

## 4 *In vitro* and *in vivo* models

Both *in vitro* and *in vivo* models have been used to assess the wound-healing properties of CNT-based materials. Fibroblasts, keratinocytes, and endothelial cells are the three most common cell types used *in vitro* to examine adhesion, proliferation, migration, and ECM deposition. CNT-containing hydrogels and scaffolds have been shown to promote fibroblast growth, collagen production, and keratinocyte migration, ultimately resulting in accelerated re-epithelialization and granulation tissue formation (Zhou et al., 2024). The electrical conductivity of CNT scaffolds has been shown to enhance gap junction activity and cell communication, particularly when combined with external electrical stimulation, thereby facilitating faster wound closure. Preclinical animal testing, particularly in diabetic rat wound models, has provided clear indications of the translational potential of CNT-based wound dressings. CNT-protein-cellulose hybrid hydrogels accelerated wound contraction and promoted tissue regeneration under NIR irradiation, yielding better healing outcomes than those of conventional controls (Tian et al., 2025). *In vitro* antibacterial assessments also show that CNT-based composites exert an efficient bactericidal effect against *S. aureus, E. coli*, MRSA, and biofilm-forming bacteria through a series of mechanisms, including physical disruption of the cell membrane, increased ROS generation, and NIR induced photothermal activity. Multifunctional CNT-derived nanocomposite hydrogels have been shown to promote rapid granulation tissue formation, angiogenesis, and re-epithelialization, in addition to sterilizing multidrug-resistant pathogens in infected wounds (Gao et al., 2025). In addition, multifunctional drug or bioactive-molecule-loaded CNT systems functionalized with agents such as silver nanoparticles have demonstrated synergistic antibacterial and regenerative activities, thereby demonstrating their dual functions of controlling infection whilst promoting tissue repair. In addition, CNT composites with stimuli-responsive elements, i.e., NIR-triggered drug release and antioxidant activities, cleared bacterial biofilms and facilitated tissue regeneration and angiogenesis in rodent wound models (Yang et al., 2025). The functionalized CNTs incorporated within a biocompatible matrix exhibited low systemic toxicity *in vivo*, highlighting their excellent safety profiles for future clinical applications. Altogether, these studies demonstrate that CNT-based wound-healing systems combine antimicrobial efficacy, immunomodulation, and regenerative assistance, representing a robust, multifunctional platform for effective wound care management (Vardharajula et al., 2012).

## 5 CNT-based materials for wound healing applications

In recent years, carbon nanotubes (CNTs) have played a pivotal role in biomedical applications, especially in wound healing. Their unique tubular shape, nanoscale rough surface, and potential to create various types of surface chemistry result in a variety of properties spanning structural, electrical, and biochemical domains. Unlike traditional “inert” dressings that passively cover and protect the wound, most carbon nanotube-based systems can actively participate in the progressive stages involved in the healing cascade. Here, we summarize recent findings by consolidating them into five application areas of wound healing: hemostasis induction, anti-infection efficacy, inflammation alleviation, angiogenesis facilitation, tissue repair stimulation, and on-site chemical changes testing. These studies collectively indicate that CNTs are promising next-generation wound-dressing platforms (Madaninasab et al., 2025).

### 5.1 Hemostatic applications of CNTs

**FIGURE 4:**
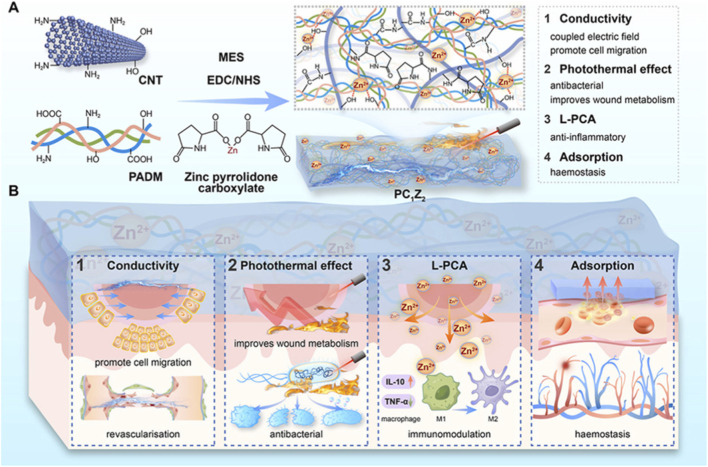
Schematic representation showing the preparation of carbon nanotube-decellularized dermal matrix sponges loaded with zinc-pyrrolidone carboxylate. **(A)** A multifunctional photothermal electroactive sponge scaffold PC_1_Z_2_ was fabricated by combining CNTs, PADM (porcine decellularized dermal matrix), and zinc pyrrolidone carboxylate. **(B)** Recovery of the wound with the unique synergistic aspects of conductivity, photothermal effect, immunomodulation, and adsorption. Reproduced with permission from Wu et al. (Wu et al., 2025).

The initial step of wound management, especially in traumatology, is rapid hemostasis. Hemorrhage continues to be the leading cause of preventable mortality, and traditional dressings such as cotton gauze remain limited to acting as blood absorbents with negligible influence on coagulation. CNT-composites, however, shift this paradigm by introducing a nanoscale architecture that promotes platelet adhesion and fibrin netting formation to facilitate coagulation. Tan et al. reported that the addition of CNTs as fillers to carbonized cellulose aerogels could convert it into a highly efficient hemostatic material (Tan et al., 2025). Porous and ultralight structures enabled rapid blood absorption, and rough nanosurfaces promoted platelet adhesion/aggregation. In both animal liver and tail bleed *in vivo* models, compared to cellulose (control), the clotting time with CNT-aerogel was shortened by 3-fold, indicating that nanoscale reinforcement is not purely mechanical but also interacts actively with coagulation. Based on the concept of multifunctionality, Wu et al. reported a CNT-based sponge scaffold incorporating pyrrolidonecarboxylic acid zinc (PC_1_Z_2_) for the therapeutic treatment of diabetic wound healing, including inflammation and infection (Wu et al., 2025), as shown in Figure 4. The CNT network served as a pro-coagulant surface, while the zinc ions exerted antimicrobial and anti-inflammatory properties. This is especially beneficial for diabetic wounds, complicated by trauma-driven infection and inflammation. They showed that combining hemostatic and antimicrobial activities in a single dressing provides dual roles, simultaneously treating multiple early wound-healing challenges. Taken together, these studies demonstrate the transition of CNT-based dressings from passive absorbents into active, multimodal systems. Towards immediate enemy action: CNTs stop immediate bleeding and early infection risks by initiating blood clotting and inhibiting bacterial growth, suggesting significant potential for use in injured patients and even in chronic wound applications.

### 5.2 Antibacterial and anti-inflammatory systems

**FIGURE 5:**
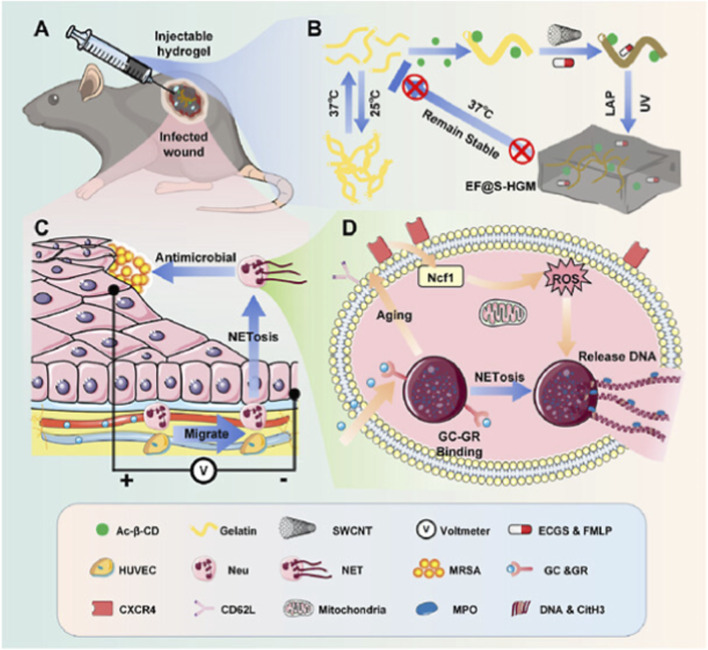
Schematic representation of nanomaterial-assisted wound healing mechanisms. **(A)** An infected wound area was treated with injectable hydrogel in mice for localized treatment. **(B)** The hydrogel is thermostable, maintaining its integrity at body temperature and enabling stable release of therapeutic cargo. **(C)** The hydrogel promotes NETosis, thereby enhancing antimicrobial activity and recruiting cells for tissue migration and healing **(D)**. Mechanistically, NETosis in neutrophils is regulated by the Ncf1 gene, GC-GR signaling, and mitochondrial ROS, thereby promoting DNA release to facilitate effective infection control. Reproduced with permission from OuYang et al. (Ou et al., 2025).

Once bleeding is stopped, wounds are at high risk of infection. Bacterial colonization delays healing and results in chronic inflammation, which may cause systemic complications. CNTs have been well established for their intrinsic antibacterial activities due to their hydrophobicity, membrane-puncturing potential, and ability to generate reactive oxygen species (ROS) (Maleki Dizaj et al., 2015). In a recent study, researchers fabricated a hydrogel-based composite system incorporating silver nanoparticles (AgNPs), MWCNTs, and Pluronic F127 to manage bacterial infection at the wound site, thereby imparting antioxidant and antimicrobial properties. The MWCNT-Ag/PF127 composites were demonstrated for enhanced antibacterial and anti-biofilm activities while maintaining a balance between bioactive properties and biocompatibility (Ryu et al., 2025). On the catalytic front, Wang et al. described hydrophobic Fe/Co-loaded CNTs (Chen et al., 2025). These composites disrupted bacterial membranes and generated ROS, resulting in effective bactericidal activity against both gram-positive and gram-negative strains. Their lipophilicity promoted close contact between bacterial membranes, thereby increasing permeabilization. Photothermal designs are another major direction for these studies. Chao et al. constructed CNT-lignin/PVA hydrogels that can induce local hyperthermia upon NIR irradiation at the wound site with the improved antibacterial activity against *E. coli* and *S. aureus* (Chao et al., 2023). *In vivo* studies demonstrated on-demand elimination of pathogen propagation at the wound site through a photothermal antibacterial effect, reduced inflammation, and rapid wound healing. Li et al. engineered CNT hydrogels exhibiting catalase and superoxide dismutase-mimicking “nanozyme” activity (Alhashmi Alamer and Almalki, 2022). These hydrogels simultaneously killed bacteria and scavenged ROS to re-establish redox homeostasis at the wound site. This dual-action system represents a significant innovation, as CNT composites can eradicate pathogens, modulate the inflammatory microenvironment, and facilitate angiogenesis. In another study, OuYang et al. introduced a carbon nanotube-based injectable nanoconductive hydrogel with a host-guest supramolecular macromolecule approach. The authors used single-walled carbon nanotubes to fabricate an endogenously electric-field-responsive hydrogel that promotes rapid migration of multiple cells at the wound site. In addition, the hydrogel was encapsulated with endothelial cell growth supplement for cell proliferation, and N-Formyl-Methionyl-Leucyl-phenylalanine to recruit neutrophils and induce Neutrophil Extracellular Traps. Altogether, the hydrogel modulates the inflammatory response, provides antimicrobial activity, and repairs wounds (Ou et al., 2025), as shown in Figure 5.

**FIGURE 6:**
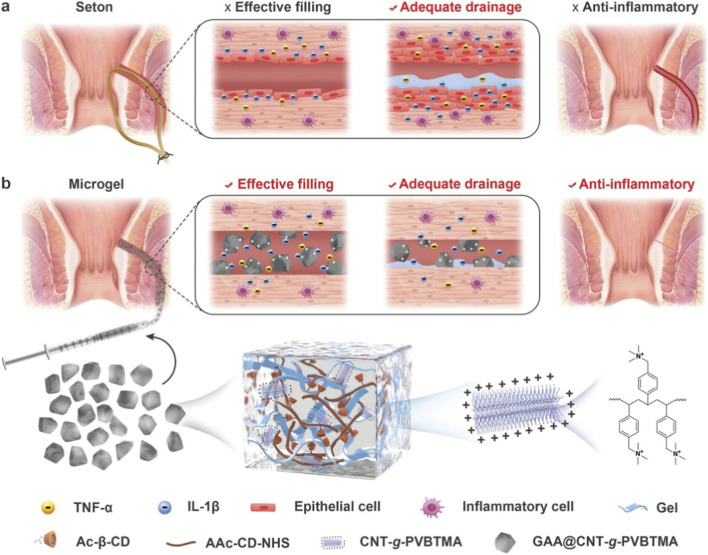
Schematic representation of the drainage and anti-inflammatory role of quaternized molecular brush-grafted injectable microgel (denoted as GAA@CNT-g-PVBTMA) in anal fistula repair. **(a)** In conventional anal seton surgery, the seton ensures continuous drainage of exudates but lacks the ability to fill the fistula tract. Exposure to ongoing inflammatory triggers and promotes aberrant epithelial growth along the fistula wall, impeding closure and healing process. **(b)** The GAA@CNT-g-PVBTMA microgel binds negatively charged inflammatory cytokines through electrostatic interactions, while simultaneously providing structural filling, controlled drainage, and anti-inflammatory activity, thereby supporting effective fistula healing. Reproduced with permission from Wang et al. (2025a).

In another example of developing advanced systems for bioelectronic stimulation, Nie et al. developed mechano-electric CNT hydrogels that provided both antimicrobial action and electrical stimulation, thereby enhancing fibroblast migration and proliferation (Nie et al., 2025). Building on this, Xu et al. created a self-powered casein-CNT “E-dressing” capable of harvesting biomechanical energy from wound movement to deliver electrical cues, while also exhibiting photothermal antibacterial activity under NIR irradiation Xu et al., 2025. In the context of electronic skin (E-skin) devices, Song et al. fabricated thermoelectric CNT-polymer composites with dual antibacterial and thermoreceptor-like activity, pointing toward integrated sensory applications with self-healing properties (Song et al., 2025). Another study reported by Xu et al. demonstrated the development of CNT-GelMA-based electroactive macroporous nanocomposite hydrogels via pickering emulsions, combining antibacterial activity with structural porosity to facilitate cellular infiltration and enhance wound-healing performance. The mechanical performance, aside from pore dimensions and conductivity, can also be tailored by varying the CNT content (Xu et al., 2020). In a study aimed at developing treatments for anal fistula, Wang et al. developed quaternized CNT molecular brush grafted injectable microgel systems to effectively fill the fistula and facilitate the drainage of exudate with antibacterial and anti-inflammatory effects, as shown in Figure 6 (Wang et al., 2025a). Related investigations also highlighted multifunctional hydrogels with bacteriostatic, self-healing, conductive, and drug-delivery properties (Zarouki et al., 2025) (Lai et al., 2025). Overall, CNTs are not limited to antibacterial action through a single mechanism but instead contribute multiple strategies, including ROS generation, photothermal conversion, enzymatic mimicry, and electrical stimulation. This versatility enables tailored dressings for both acute trauma and chronic infection, providing a robust foundation for future multimodal infection-control systems.

### 5.3 CNTs in diabetic wound repair and angiogenesis

**FIGURE 7:**
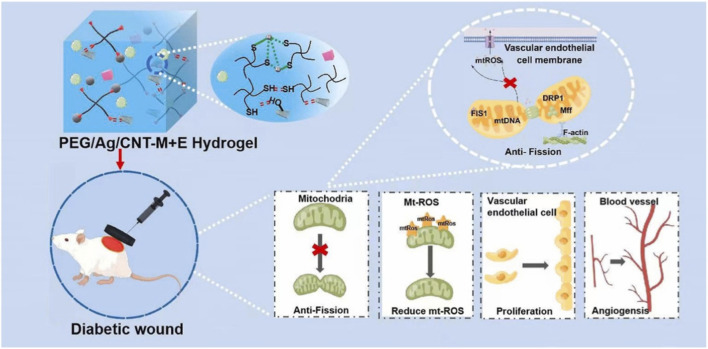
PEG/Ag/CNT-M + E hydrogel (polyethylene glycol/silver/carbon nanotube-metformin and exosome) enhances diabetic wound healing by inhibiting mitochondrial fission (anti-fission), reducing mitochondrial reactive oxygen species, promoting vascular endothelial cell (VEC) proliferation, and stimulating angiogenesis. Reproduced with permission from Zhang et al. (2023).

Diabetic ulcers are one such clinical challenge, where the wound is usually impaired due to angiogenesis deficiency, mitochondrial dysfunction, and inflammation. CNT-based materials have been used to address these problems by combining bioactive delivery systems with conductive scaffolds. Zhang et al. synthesized an exosome/metformin self-healing conductive hydrogel using MWCNTs to promote chronic diabetic wound healing by interfering with mitochondrial fission (Zhang et al., 2023). The synthesized CNT-based injectable hydrogel sustained the release of exosomes and metformin, attenuated mitochondrial fission, normalized endothelial dysfunction, and induced angiogenesis, thereby interfering with the pathogenesis of diabetic ulcers at both the level of metabolic control and conductive signalling, as shown in Figure 7. In another study, Khalid et al. designed CNT-functionalized bacterial cellulose biomaterials to enhance fibroblast migration and angiogenic responses, thereby expediting the healing process in a diabetic wound model (Khalid et al., 2022). Tavakoli et al. employed a tri-layer CNT sponge scaffold incorporating insulin-like growth factor-1 for sustained delivery, to promote prolonged angiogenic signalling, durable vascular remodelling, and a faster rate of wound closure (Tavakoli et al., 2023). Recently, Naik et al. developed CNT-protein-cellulose-based conductive hydrogels for photothermal therapy and demonstrated *in vivo* photoacoustic imaging in a diabetic disease model (Naik et al., 2025). Here, the dual-purpose platform facilitated significantly faster, more effective wound closure while simultaneously monitoring vascular progression non-invasively, representing a significant breakthrough in diabetic wound care. These studies demonstrate the multifunctional potential of CNT composites, where conductivity, structural reinforcement, and therapeutic delivery converge. Such systems hold considerable promise for diabetic wound healing as they enhance angiogenesis, correct endothelial dysfunction, and stimulate fibroblast activity.

### 5.4 Tissue engineering and regenerative scaffolds

**FIGURE 8:**
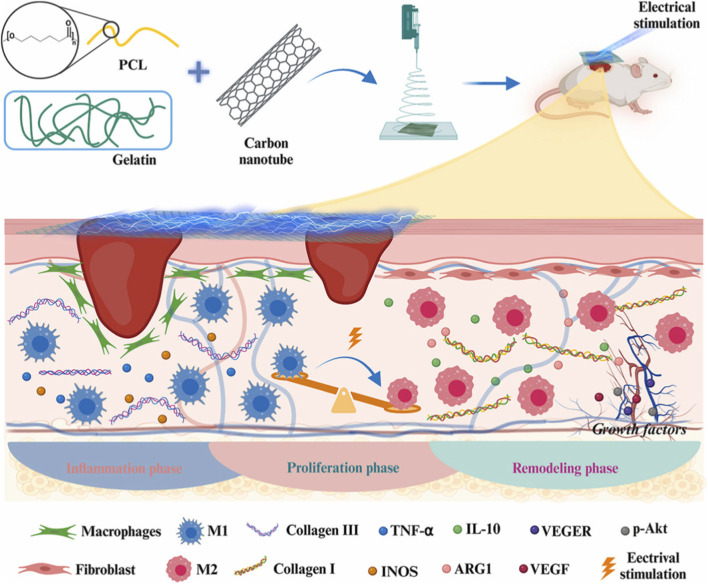
Schematic representation of the fabrication of the PCL/GE/CNT electroactive scaffold based on CNTs using the electrospinning method and combined with electrical stimulation (ES) for skin repair. By differentiating macrophages, fibroblasts, and endothelial cells, the scaffold with local ES supports wound healing through early remodelling of the inflammatory microenvironment and by inducing re-epithelialization and collagen deposition. Reproduced with permission from (Chen et al., 2025).

**FIGURE 9:**
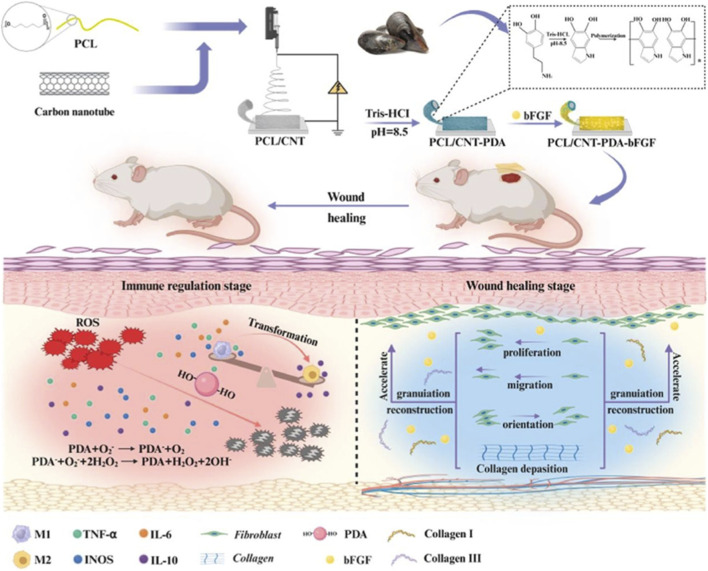
The first row shows the schematic of scaffold fabrication, starting from PCL (polycaprolactone) and CNT, followed by PDA (polydopamine) coating and bFGF (basic fibroblast growth factor) loading, and application to a murine wound model. The second row shows the immune regulation stage, with PDA reducing ROS (reactive oxygen species) and transforming macrophages from M1 (pro-inflammatory) to M2 (anti-inflammatory) phenotypes, whereas in the wound-healing stage, bFGF accelerates fibroblast proliferation, migration, granulation, and collagen (Col I/III) deposition. Reproduced with permission from (Cui et al., 2024).

Apart from treatment for diabetic-related diseases, CNTs have been incorporated into regenerative scaffolds to enhance fibroblast activity, keratinocyte migration, stem cell therapies, and drug delivery. CNTs’ conductivity enables scaffolds to transmit bioelectric signals, while mechanical reinforcement improves structural stability. Liu et al. demonstrated that chitosan/gelatin/MWCNT scaffolds under electrical stimulation show enhanced fibroblast proliferation and elevated expression of type I and type III collagen compared with non-conductive counterparts (Liu et al., 2025). Forero-Doria et al. reported supramolecular CNT-cellulose hydrogels for sustained release of bioactive compounds with antimicrobial and *in vivo* wound-healing regenerative benefits (Forero-Doria et al., 2020). Wang et al. reported cellulose/polypyrrole/CNT-based electroactive hydrogels for enhanced cell proliferation under electrical stimulation in wound healing (Wang et al., 2020). Li et al. expanded the functionality of CNTs by fabricating self-healing hydrogels that retain conductivity and mechanical integrity under repeated deformation (Li et al., 2020). Recently, Chen et al.'s research group constructed an electroactive, oriented wound dressing using a CNT-aligned nanofibrous sheet, which provides directional cues for fibroblast and endothelial cell proliferation and migration, thereby accelerating the healing process (Chen et al., 2025). Figure 8 represents the fabrication of PCL/Gelatin/CNT scaffolds via electrospinning to provide electrostimulation (ES) and regulate the immune microenvironment. CNTs also provide conductivity, enabling the coupling of surrounding and applied electric fields to regulate macrophage phenotypes from M1 to M2 and promote the fibroblast and endothelial cell migration. *In vivo* studies showed that PCL/Gelatin/CNT scaffolds treated with electrical stimulation inhibited the early inflammation with increased angiogenesis and collagen deposition. Such dressing provides an efficient ES-assisted method for wound healing. In another report, Hashemi et al. and the research group seeded human Wharton’s jelly stem cells tagged with superparamagnetic iron oxide nanoparticles onto PVA/chitosan/CNT scaffolds to study their effect on burn wounds. The outcomes of their study include enhanced healing of burn wounds and MRI monitoring of transplanted cells, highlighting their clinical relevance (Hashemi et al., 2025). In the research reported by Cui et al., an electrospun PCL/CNT fibrous scaffold system was coated with polydopamine containing basic fibroblast growth factor (bFGF) to promote tissue regeneration (Cui et al., 2024). Encapsulation of CNTs modulates the immune microenvironment by promoting macrophage polarization from M1 to M2. As shown in Figure 9, the electroactive aligned fibres can promote fibroblast proliferation and guide cell assembly. *In vitro* and *in vivo* studies showed decreased inflammation, enhanced granulation tissue, increased collagen deposition, and re-epithelialization. Altogether, these studies portray CNT scaffolds as bioelectronic regenerative platforms that integrate cell guidance, drug delivery, and self-healing functions. They are versatile enough to support both routine wound closure and advanced strategies such as stem cell transplantation.

### 5.5 Smart CNT dressings, sensors, and burn management

Recent advancements towards wound management include the development of innovative dressings that integrate therapeutic and sensing capabilities. CNTs’ conductivity, flexibility, and photothermal properties position them as prime candidates for bioelectronic skins and responsive hydrogel systems. Recently, Ji et al. synthesized CNT-based hydrogel bandage containing glucose and pH sensors for non-invasive monitoring of diabetic wounds (Ji et al., 2025). Liu et al. fabricated an electronic skin scaffold with therapeutic and diagnostic capabilities (Liu et al., 2023). Shen et al. reported a CNT-based nanocomposite conductive hydrogel for flexible monitoring of human motion in daily life and for detecting local temperature changes to monitor the wound-healing process (Shen et al., 2023). Song et al. developed a temperature-responsive electronic platform using a CNT-based thermoelectric polymer composite with self-healing and stretchable properties (Song et al., 2025). Shi et al. have fabricated a novel CNT-based hydrogel with heat-storage capacity, thermal-conductivity, and adhesive ability properties. Thus, CNTs are a potential candidate for burn therapy, laser treatment, and heat-protective clothing (Shi et al., 2022). Pantzke et al. reported that CNTs can modulate interactions between CNTs and fibers in the lungs of patients with pulmonary fibrosis, highlighting the importance of detailed long-term biosafety assessment as CNT-based dressings approach clinical use (Pantzke et al., 2023). Together, these intelligent systems exemplify the progression of CNTs applications: active dressings capable of sensing, stimulating, and healing in real time, accompanied by safety assessments to ensure clinical translation.

## 6 Clinical translation and challenges

The translation of carbon nanotube (CNTs)-based materials from preclinical research to clinical practice holds immense potential to revolutionize wound healing, particularly for chronic wounds such as diabetic ulcers, venous leg ulcers, and burns. However, significant hurdles remain in achieving regulatory approval, ensuring safety, and scaling production. This section outlines the current state of clinical translation, key challenges, and strategies to overcome them, drawing on recent studies and patents from 2020 to 2025.

### 6.1 Current progress in clinical translation

CNT-based wound healing materials have shown promise in preclinical models, with applications ranging from antimicrobial dressings to regenerative scaffolds. For instance, bacterial cellulose/MWCNT films achieved 99% wound closure in diabetic rat models within 21 days (Khalid et al., 2022), and CNT/GelMA demonstrated rapid hemostasis and sustained drug release in murine wounds (Li et al., 2022a) (Zhao et al., 2018). Early-phase clinical trials, primarily conducted in Asia and Europe, are largely centered on evaluating CNT-hydrogel composites for use in burn and surgical wounds, with a focus on safety and efficacy. Patents from 2023 to 2024 describe CNT-based dressings with integrated sensors for real-time wound monitoring, indicating industry interest in clinical applications (Hughes et al., 2024). However, no CNT-based wound healing product has yet received widespread regulatory approval (e.g., FDA or EMA), reflecting the nascent stage of translation.

Toxicity Concerns: A primary barrier is the potential cytotoxicity of CNTs, particularly pristine forms, which can induce inflammation, oxidative stress, or genotoxicity due to impurities (e.g., metal catalysts) or aggregation. Long-term bioaccumulation of non-biodegradable CNTs, especially MWCNTs, raises concerns about systemic toxicity, as they may persist in tissues or translocate to organs like the lungs or liver (Cheng et al., 2022). Functionalization with biocompatible groups (e.g., PEG, carboxyl) mitigates these risks; however, studies show variable biocompatibility depending on the CNT type, size, and dose. For example, high-dose SWCNTs (>1 mg/mL) caused fibroblast apoptosis *in vitro*, while low-dose functionalized CNTs (0.1–0.5 wt%) were well-tolerated (Fraser et al., 2020). Comprehensive toxicological profiling in complex models (e.g., porcine or humanized systems) is needed to establish safe exposure limits (Sayed and Shaikh, 2024).

Scalability and Cost: Producing high-purity CNTs at scale remains costly, with prices ranging from $50 to $ 500/g for biomedical-grade materials. Synthesis methods, such as chemical vapor deposition (CVD), require expensive catalysts and energy-intensive processes, while purification to remove impurities adds complexity. Scaling fabrication of CNTs composites, such as hydrogels or electrospun mats, is challenging due to difficulties in achieving uniform CNT dispersion and reproducibility. These factors limit cost-effectiveness, a critical consideration for widespread clinical adoption, especially in resource-limited settings (Madikere Raghunatha Reddy et al., 2025).

Regulatory Hurdles: Regulatory and translational challenges remain major hurdles for bringing CNT-based wound dressings into clinical use. Regulatory bodies require extensive, continuous data on biocompatibility, biodegradation, systemic distribution, and manufacturing consistency. However, CNT materials are intrinsically heterogeneous, varying in diameter, length, chirality, purity (metallic or semiconducting), surface chemistry, and degree of functionalization, which complicates the establishment of uniform toxicity tests and consistent batch-to-batch standards. This variability can lead to impurity-mediated cytotoxicity, endotoxin contamination, and unpredictable inflammatory responses.

Current nanomaterial standards, such as ISO/TS 80004, provide only high-level definitions and lack CNTs-specific protocols for assessing safety, degradation kinetics, sterility assurance, or long-term tissue retention. As a result, regulators must evaluate CNT-based devices individually, resulting in slowing the review process and hindering industrial translation. The clinical pipeline faces similar constraints, with a scarcity of long-term preclinical studies and an almost complete absence of randomized controlled clinical trials. Without robust human data in chronic wounds, burns, or diabetic ulcers, CNT-based dressings remain confined to laboratory and small-animal research.

Strategies to overcome challenges: Advances in purification techniques, such as acid washing and ultracentrifugation, reduce impurities, while biodegradable CNT derivatives (e.g., carboxylate CNTs) minimize bioaccumulation risks. Developing cost-effective synthesis methods, such as plasma-enhanced CVD, and scalable fabrication techniques (e.g., automated 3D printing) could lower costs. Collaborative efforts between academia, industry, and regulators are essential to establish standardized testing protocols and accelerate clinical trials. Emerging translational platforms, including injectable hydrogels, microneedle patches, and 3D-printed scaffolds, offer scalable, multifunctional strategies for clinical deployment. These approaches emphasize integrating nanomaterials with imaging-guided and precision-medicine paradigms.

## 7 Future perspectives

The future direction of CNTs for wound-healing materials is to establish multifunctional, patient-specific platforms utilising advanced technologies to overcome the above limitations. The key directions include biodegradable CNTs, novel wound dressings, and possible synergy with new intervention(s). Biodegradable CNTs prepared by oxidative cleavage or enzymatic degradation could minimise long-term toxicity and allow safe elimination from the body. Studies of graphite oxides may guide the development of CNTs. Innovative dressings with embedded sensors to monitor pH, temperature, or markers of infection, combined with AI-based analytics, herald personalised therapy. For example, a 2024 patent introduced a real-time monitor and NIR-triggered drug-release CNT hydrogel for adaptive treatment. Combining CNTs with gene therapy (e.g., siRNA delivery) or stem cell scaffolding may improve regeneration, particularly in chronic wounds. Technical advances in 3D bioprinting and nanotechnology may enable the fabrication of customised scaffolds for multidimensional wounds in the future, and multicentre clinical studies with large sample sizes will further verify their therapeutic effects. Shared efforts to lower costs and normalise regulation will be key to commercialisation, which could revolutionise wound care over the next decade.

## 8 Conclusion

CNTs have tremendous potential for wound healing due to their distinctive mechanical, electrical, and thermal properties, enabling the fabrication of advanced wound dressings that address infection and inflammation and facilitate tissue regeneration. To maintain a broad perspective, Table 1 presents CNT-based wound-healing approaches, including the mechanisms and primary outcomes discussed above. This integrative map makes evident that CNT composites encompass haemostasis, infection prevention, immunomodulation, angiogenesis, and regenerative scaffolding. Functionalized CNT-based platforms, such as hydrogel scaffolds, have shown preclinical efficacy, and several of these studies are now entering early-phase clinical trials. However, these cellular materials can only be further developed into viable products after toxicity and scale-up are addressed through improved purification strategies for purity, biodegradable system design, and standardisation. Over time, innovative wound treatments, whether that be smart systems, AI, or regenerative therapies, will revolutionise the treatment of chronic wounds, resulting in better patient outcomes and reduced healthcare burdens.

**TABLE 1:** Summary of carbon nanotube (CNT)-based wound healing studies highlighting material systems, mechanisms, and outcomes.

Category	CNT system/Composite	Key mechanism(s)	Model/Outcome	Ref. no.
Hemostatic	CNT-cellulose aerogel	Nanosurface promotes platelet adhesion, fibrin mesh	*In vivo* liver and tail bleeding leads to 3× faster clotting	Tan et al. (2025)
Hemostatic	CNT-zinc sponge	CNT as pro-coagulant surface; Zn^2+^ as antimicrobial and anti-inflammatory	Diabetic wounds: ↓IL-6/TNF-α, ↑VEGF/TGF-β1, enhanced neovascularization	Wu et al. (2025)
Antibacterial/Anti-inflammatory	Fe/Co-loaded CNTs	Hydrophobic, ROS generation, bacterial membrane disruption	Broad-spectrum antibacterial	Wang et al. (2025b)
Antibacterial/Anti-inflammatory	CNT-lignin/PVA hydrogel	NIR photothermal antibacterial; adaptable elastic hydrogel	Pathogen eradication under NIR	Chao et al. (2023)
Antibacterial/Anti-inflammatory	molybdenum disulfide nanosheet/CNT “nanozyme” hydrogel	Catalase/SOD mimicry, ROS scavenging	Bactericidal + redox homeostasis	Li et al. (2022b)
Antibacterial/Anti-inflammatory	Injectable conductive CNT hydrogel + EGF factors	Neutrophil recruitment, NETs, vascular remodeling	Infected wound healing	Ou et al. (2025)
Antibacterial/Anti-inflammatory	Zn NPs/CNTs/PNIPAM	Electrical stimulation + antimicrobial	Enhanced fibroblast migration and proliferation	Nie et al. (2025)
Antibacterial/Anti-inflammatory	Casein-CNT “E-dressing”	Self-powered, NIR photothermal + electrical cues	Chronic wound management	Xu et al. (2025)
Antibacterial/Anti-inflammatory	Thermoelectric CNT-polymer composites	Thermoreceptor-like + antibacterial	Integrated sensory dressings	Song et al. (2025)
Antibacterial/Anti-inflammatory	Macroporous CNT/gelatin methacryloyl. hydrogels	Porosity + antibacterial	Cellular infiltration + antibacterial	Xu et al. (2020)
Antibacterial/Anti-inflammatory	Quaternized CNT microgels	Injectable, drainage + antibacterial	Anal fistula repair, anti-inflammatory	Wang et al. (2025a)
Diabetic wound healing	CNT–protein–cellulose conductive hydrogel	Photothermal therapy + PA imaging	Diabetic rat: closure + vascular monitoring	Naik et al. (2025)
Diabetic wound healing	CNT hydrogel + exosomes and metformin	Suppressed mitochondrial fission, corrected endothelial dysfunction	Enhanced angiogenesis in diabetic ulcers	Zhang et al. (2023)
Diabetic wound healing	CNT-functionalized bacterial cellulose	Enhanced fibroblast migration + angiogenic response	Faster closure in diabetic wounds	Khalid et al. (2022)
Diabetic wound healing	polycaprolactone/gelatin/CNT	Increased expression of Col I/III and VEGF, angiogenesis, vascular remodeling	efficient wound repair	Chen et al. (2025)
Diabetic wound healing	Photodetachable CNT-PC1Z2 scaffold	Zn^2+^ release + NIR antibacterial + angiogenesis	Diabetic rats: 98% antibacterial efficiency, faster closure	Wu et al. (2025)
Regenerative scaffolds	CS/Gel/MWCNTs conductive scaffold	Electrical stimulation→ fibroblast proliferation, collagen synthesis	Enhanced tissue regeneration	Liu et al. (2025)
Regenerative scaffolds	CNT-aligned nanofibrous sheet	Provides directional keratinocyte migration	Accelerated re-epithelialization	Chen et al. (2025)
Regenerative scaffolds	superparamagnetic iron oxide nanoparticles/CNT/PVA/chitosan scaffold with Wharton’s jelly stem cells	Stem cell therapy + MRI monitoring	Burn wound repair + stem cell tracking	Hashemi et al. (2025)
Regenerative scaffolds	CNT–cellulose supramolecular hydrogel	Sustained drug release, antimicrobial, regenerative	Multifunctional wound healing	Forero-Doria et al. (2020)
Regenerative scaffolds	bacterial cellulose/polypyrrole/carbon nanotube	Electroactive stimulation	Enhanced fibroblast proliferation	Wang et al. (2020)
Smart dressings/sensors	PVA/CNT-hydrogel bandage with glucose/pH sensors	Real-time wound monitoring	Non-invasive diabetic wound monitoring	Ji et al. (2025)
Smart dressings/sensors	acellular dermal matrix/CNT “E-skin” scaffold	Integrated therapeutic + diagnostic	Versatile wound bioelectronics	Liu et al. (2023)
Smart dressings/sensors	CNT nanocomposite hydrogel	Robust elasticity + multifunctional sensing	Wearable wound monitoring	Shen et al. (2023)
Smart dressings/sensors	Thermoelectric CNT composite	Thermoreceptor + antibacterial	Sensory dressing with antibacterial	Song et al. (2025)
Burn management	thermally responsive PEG derivatives/CNT-hydrogel dressings	Thermal conductivity + heat storage	Improved cooling in burn therapy	Shi et al. (2022)
Burn management	CNT/gelatin methacryloyl macroporous nanocomposite hydrogels	Antibacterial + regenerative	High-exudate wound healing	Xu et al. (2020)
Safety/biosafety	CNT-fiber pulmonary model	CNT–fiber interaction, fibrosis modeling	Long-term biosafety concerns	Pantzke et al. (2023)
